# The influence of fasting and carbohydrate-enriched drink administration on body water amount and distribution: a volunteer randomized study

**DOI:** 10.1186/s13741-021-00198-0

**Published:** 2021-08-10

**Authors:** Jakub Kukliński, Karol P. Steckiewicz, Bartosz Sekuła, Aleksander Aszkiełowicz, Radosław Owczuk

**Affiliations:** 1 Student Scientific Society, Department of Anesthesiology and Intensive Care, Faculty of Medicine, Medical University of Gdansk, Gdansk, Poland; 2 Department of Anesthesiology and Intensive Care, Faculty of Medicine, Medical University of Gdansk, Gdansk, Poland

**Keywords:** Fasting, Bioelectrical impedance analysis, Total body water, Pre-op, Perioperative patient management, Enhanced recovery after surgery (ERAS), Intracellular water, Extracellular water, Dehydration

## Abstract

**Background:**

Fasting prior to anesthesia is considered aspiration prophylaxis. However, prolonged food and drink restrictions may increase the risk of other complications. The aim of this study was to assess whether a carbohydrate-enriched drink (Nutricia™ preOp®), recommended by the enhanced recovery after surgery (ERAS) protocol, can improve body hydration in fasting healthy individuals.

**Methods:**

Measurements were done with the bioelectric impedance analysis with a Fresenius body composition monitor. Body composition, total body water, water distribution, and hemodynamic parameters were measured at the beginning of the study and after 10 h and 12 h of fasting. Patients fasted for 10 h and then were divided into two groups: the control (*n* = 40) and the pre-op group (*n* = 41). The pre-op group received 400 mL of Nutricia™ preOp®, as suggested in the ERAS guidance. The two-tailed Student’s t test was used to compare two groups with normally distributed data and homogenous variances; if variances were heterogeneous, Welch’s test was used. The Mann-Whitney U test was used to compare two groups with non-normal data distribution. *p* < 0.05 was considered statistically significant.

**Results:**

We found no significant differences between the control and pre-op groups regarding body water distribution and body composition. We did not observe significant losses in the total body water after fasting. Also, blood pressure was not affected by fasting.

**Conclusion:**

We have proven that pre-op did not impact either body composition or body water.

**Trial registration:**

ClinicalTrials.gov, NCT04665349. Registered on 11 December 2020—retrospectively registered.

## Introduction

The current American Society of Anesthesiologists (ASA) guidelines recommend that patients should fast for 6 h and refrain from drinking clear liquids for 2 h before elective surgery (Warner et al. 1999). Excessive fasting is not recommended; however, in many hospitals, patients are required to not eat from the evening before surgery (Chin et al. 2006). Even short-term fasting causes insulin resistance, which leads to hyperglycemia and increases the risk of complications in the postoperative period, and lowers the level of insulin-like growth factor (IGF-1), which impairs wound healing (Nygren 2006). This is particularly disadvantageous because the stress response to surgical injury has similar metabolic effects to starvation (Nygren 2006). Moreover, withholding liquid administration may cause dehydration in patients, which increases the risk of hypotension during induction of anesthesia. Unfortunately, direct monitoring of hydration is impossible in the operating room, and only indirect hemodynamic parameters may be used to estimate patient hydration (Pang et al. 2019; Szczepańska et al. 2020). On the other hand, food and fluid restriction causes the stomach to empty, reducing the risk of pulmonary aspiration of gastric contents. It was proven that ≥ 1 mL kg^−1^ of fluid in the stomach may cause a clinically significant risk of aspiration (Bouvet et al. 2019). Thus, intravenous liquids are administered.

There is no consensus on perioperative fluid management among anesthesia providers (Jacob et al. 2008). It is clear that crystalloids are superior to colloids in perioperative fluid therapy (Jacob et al. 2008). In the past, 5% glucose and 0.9% sodium chloride solution (named normal saline) were commonly used. It was suggested that 5% glucose may be beneficial in overcoming insulin resistance, and 5% glucose was thought to increase intracellular water content; however, these speculations were never confirmed (Chin et al. 2006). Due to their significant disadvantages, these fluids lost their primacy to balanced crystalloids. Although normal saline is isotonic, it is considered a non-balanced crystalloid (Corrêa et al. 2015). It has 154 mEq L^−1^ of chloride, which is 1.5 times higher than the level in human serum (Corrêa et al. 2015). Additionally, the strong ion difference (SID) of normal saline is much lower than that of serum: 0 mEq L^−1^ vs. 40 mEq L^−1^ (Corrêa et al. 2015). In summary, a large volume infusion of normal saline will reduce SID and may cause hyperchloremic acidosis (Corrêa et al. 2015). However, 5% glucose is an isotonic solution, glucose is rapidly metabolized to water and carbon dioxide after intravenous administration (Chin et al. 2006). Thus, administration of 5% glucose is equal to the administration of pure water.

Therefore, the enhanced recovery after surgery (ERAS) protocol has been established to increase patient’s well-being after surgery. One of the important aspects of ERAS is rational fluid and food restriction prior to anesthesia (Borys et al. 2020; Kaye et al. 2020; Taniguchi et al. 2012). Both ERAS and the European Society for Clinical Nutrition and Metabolism (ESPEN) guidelines recommend oral intake of a carbohydrate-rich drink prior to surgery (Nygren et al. 2013; Weimann et al. 2017).

The aim of this study was to assess whether a carbohydrate-enriched drink (Nutricia^TM^ preOp®), recommended in ERAS protocols improves body hydration in fasting healthy individuals. We hypothesized that pre-op would improve body hydration and will not impact body composition. Measurements were done with the bioelectric impedance analysis. To the best of our knowledge, this is the first study of the kind.

## Materials and methods

The study was conducted as a single-center randomized controlled open-label study with balanced randomization conducted in Poland. Study protocol was accepted by Independent Bioethics Committee for Scientific Research at Medical University of Gdansk (resolution 126/2014, from 27th May 2014). The study was carried out according to Good Clinical Practice Guidance (GCP), all precipitants signed written consent. The study took place at the Department of Anesthesiology and Intensive Care of Medical University of Gdansk, Gdansk, Poland, from September 2019 to October 2020. Study design does not contain follow-up. Full study protocol is available from the corresponding author upon request. Study was retrospectively registered at ClinicalTrials.gov (NCT04665349) at 11 December 2020

### Participants

Following approval by the institutional ethics committee and obtaining written informed consent, we recruited 81 adult volunteers of ASA physical status 1 or 2. The study was performed on healthy individuals. The exclusion criteria were chronic kidney disease, heart failure, phenylketonuria, episodes of hypoglycemia, or other carbohydrate metabolism disorders. The first measurements were taken at 8:00 pm. Body composition was measured in the supine position using two sets of electrodes for unilateral hand and foot measurements. Body mass and blood pressure were also measured. Participants were asked to abstain from food for the next 10 h. They were allowed to drink clear liquids for the next 2 h, after which they had to abstain from all liquids. Second measurements were taken at 6:00 am. Then, participants were divided into two groups, the control and pre-op group, using a computer-generated randomization plan (www.randomization.com). Allocation ratio was 1:1. The control group was not allowed to drink for the next 2 hours, while the pre-op group was given 400 mL of Nutricia™ preOp®. Both groups had to refrain from eating and drinking for the next 2 h. The final measurements were taken at 8:00 am, concluding a 12-h fasting period. Due to lack of norms for body water distribution parameters we were unable to calculate groups sizes ex ante*.*

### Bioelectrical impedance analysis

Body composition was measured using a Body Composition Monitor (Fresenius Medical Care AG & Co. KGaA, Germany), which uses non-invasive bioimpedance spectroscopy techniques (Kyle et al. 2004a; Kyle et al. 2004b). Electrodes were placed on the extremities, and an alternating current was applied. High-frequency current penetrates cell membranes, while low-frequency current does not. This phenomenon allowed the measurement of electrical resistances of total body water (TBW) and extracellular water (ECW). Those values were then used to calculate clinically relevant parameters, such as ECW, TBW, intracellular water (ICW), adipose tissue mass (ATM), and lean tissue mass (LTM) using two advanced physiological models. All output parameters were validated against reference methods.

### Carbohydrate drink

Nutricia™ preOp® is a 0.5-kcal mL^−1^, clear, carbohydrate drink for patients undergoing elective surgery. All of its energy content comes from carbohydrates, namely, maltodextrin and fructose. A 400 mL serving contains 50.4 g of carbohydrates, which is more than the 45 g recommended by the enhanced recovery after surgery (ERAS) protocol. The drink is isosmotic, with an osmolarity of 240 mOsmol L^−1^. It contains the following micro- and macro-elements per 100 mL: 50 mg of sodium, 122 mg of potassium, 6 mg of chloride, 6 mg of calcium, 1 mg of phosphate, and 1 mg of magnesium.

### Statistical analysis

No interim analyses for efficacy or futility were done. The primary endpoint was changes in the extracellular to intercellular water and the amount of total body water. Outcomes were measured after the study has ended.

Categorical variables are reported by the number and percentage of patients in each category. Continuous variables with a normal probability distribution are presented as the arithmetic mean with standard deviation. For the continuous variables with a different probability distribution, the median and the interquartile range (IQR) are given.

Fisher’s exact test was used for the comparison of categorical data. The D’Agostino & Pearson test was used to assess the normality of the data. For variables with a normal distribution, parametric tests were used; if the normality of the distribution was not confirmed, non-parametric tests were used. The two-tailed Student’s t test was used to compare two groups with normally distributed data and homogenous variances; if variances were heterogeneous, Welch’s test was used. The Mann-Whitney U test was used to compare two groups with non-normal data distribution. *p* < 0.05 was considered statistically significant.

Data were analyzed with Prism 8 software (GraphPad, USA).

## Results

### Participant characteristics

Eighty-one participants were recruited into the study, and all of them completed the study protocol. Forty participants were randomized into the control group, and forty-one people received carbohydrate drink after 10 h of fasting. There were no significant differences between groups (Table 1).

**Table 1:** Patient characteristics at the beginning of the study. Values are number [%], median (IQR [range]), or mean (SD)

Variable	Control (***n*** = 40)	Pre-op (***n*** = 41)	***p***
Female	22 [55%]	25 [61%]	0.67
Age (years)	24.5 [23.3–26.0]	25 [24.0–28.0]	0.069
Height (cm)	171.2 (9.3)	171.7 (8.7)	0.82
Body mass (kg)	67.0 (13.9)	66.8 (9.8)	0.93

### Hemodynamic parameters of the participants

There were no significant differences between systolic blood pressure (SBP) and diastolic blood pressure (DPB) at any of the time points (Table 2). We observed a significant difference in heart rate (HR) between the control and the pre-op group after 12 h of fasting (*p* = 0.0271). HR was higher in the pre-op group (Table 2).

**Table 2:** Comparison of blood pressure and heart rate between groups. Values are median (IQR [range]) or mean (SD)

Variable	0 h	10 h	12 h		***p***
			Control (*n* = 40)	Pre-op (*n* = 41)	
SBP (mmHg)	123 [115.5–135]	117 [108–125]	117.5 [103.8–125.8]	114 [105.6–127.0]	0.77
DBP (mmHg)	79 [73.5–86.0]	77 [71.5–81.5]	75 [71.0–80.8]	75 [69.5–82.0]	0.67
HR (bmp)	78 [70.0–84.0]	71 [64.0–78.5]	66.5 [59.0–74.0]	72 [66.0–79.0]	**0.03**

### Body composition of the participants

There were no significant differences between any measured parameters at the 0-hour and 10-hour time points. After randomization and carbohydrate-enriched drink administration, there were no significant differences between the pre-op and control group (Table 3).

**Table 3:** Comparison of body composition between groups. Values are median (IQR [range]) or mean (SD)

Variable	0 h	10 h	12 h		***p*** (control vs pre-op)
			Control (*n* = 40)	Pre-op (*n* = 41)	
BMI (kg m^−2^)	22.6 (2.8)	22.4 (2.7)	22.3 (2.9)	22.4 (2.5)	0.90
LTI (kg m^−2^)	14.5 [12.4–17.7]	14.3 [12.5–17.1]	15.3 [13.1–17.1]	13.4 [12.1–17.8]	0.23
LTM (kg)	41.1 [33.8–56.0]	41.1 [33.4–54.8]	43.5 [34.0–55.3]	40.2 [32.9–55.2]	0.43
Fat (kg)	15.5 [11.7–21.3]	15.3 [11.3–20.5]	13.9 [11.2–20.5]	17.3 [11.4–21.1]	0.21
FTI (kg m^−2^)	7.2 [5.8–9.5]	7.1 [5.5–9.9]	6.75 [5.5–9.4]	7.7 [5.6–10.2]	0.30
ATM (kg)	20.6 [15.9–23.4]	20.9 [15.3–27.9]	18.9 [15.3–27.9]	23.5 [15.5–28.7]	0.22
BCM (kg)	25.3 (8.3)	24.6 (8.1)	25.7 (8.5)	24.1 (8.4)	0.39

### Body water distribution of the participants

**Fig. 1:**
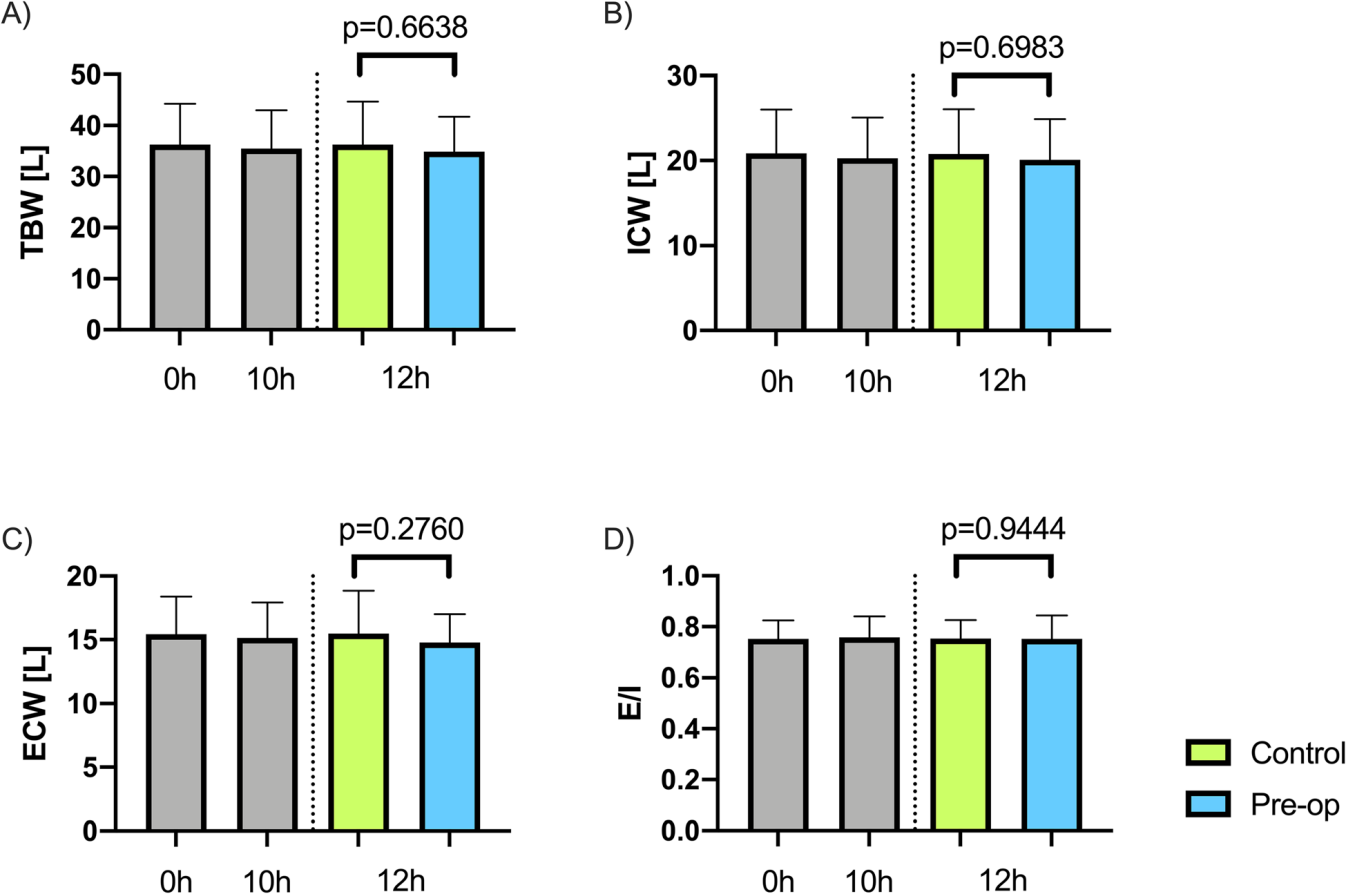
Comparison of body water distribution between groups. **A** Total body water. **B** Intracellular water. **C** Extracellular water. **D** Extracellular to intracellular water ratio

There were no significant differences between body water distribution at the 0-hour and 10-hour time points. After randomization and carbohydrate-enriched drink administration, there were no significant differences between the pre-op and control group (Fig. 1). We did not observe significant dehydration of participants over the course of the study.

## Discussion

We aimed to assess whether the administration of a carbohydrate-enriched drink impacts body water distribution in healthy fasting individuals in this study. Measurements were made with the bioelectrical impedance analysis, which is commonly used for such purposes (Kyle et al. 2004a; Kyle et al. 2004b; Song et al. 2017; Taniguchi et al. 2012; Tsukamoto et al. 2017). Participants were fasted for 10 h and the randomized into two groups: the control group, which fasted for 2 more hours, and the pre-op group, which received-carbohydrate enriched drink. The baseline hydration of our participants is worth emphasizing, as the majority of the population had E/I ratio values on the higher side of values expected for young adults (Gligoroska et al. 2016). We found no significant differences between the control and pre-op groups regarding body water distribution and body composition. We did not observe significant losses in TBW after fasting.

Other studies also aimed to understand the impact of fasting on body water distribution. Tsukamoto et al. [17] found that there were no differences in TBW, ECW, and ICW in patients with different perioperative fasting periods. In contrast, Taniguchi et al. found that patients with a shortened perioperative fasting time had a smaller decrease in TBW than patients with conventional fasting time (Taniguchi et al. 2012).

Although the carbohydrate drink did not cause any changes in water distribution, there are detrimental metabolic effects of fasting on surgery (Nygren 2006), such as insulin resistance (Soop et al. 2004) and muscle loss (Yuill et al. 2005), that can be alleviated with carbohydrate treatment. Those are associated with prolonged hospital stay, which can be shortened with carbohydrate treatment (Smith et al. 2014). Other beneficial effects include reduction of thirst, hunger, and anxiety (Hausel et al. 2001).

This study has the following limitations. First, we had no actual control of participants’ food and fluid intake and had to rely on their compliance, which may have caused alterations in fasting time. Second, we did not forbid smoking; while nicotine causes the release of antidiuretic hormone (Burn 1951), it has no effect on TBW (Vio et al. 1995). Last, we did not measure urine volume. We were unable to perform the power analysis. Ex ante analysis requires precise defined norms for the parameters, and post hoc analysis is biased.

Further studies should focus on the metabolic effects of preoperative carbohydrate treatment, the value of carbohydrates other than maltodextrin, and different routes of administration.

## Conclusion

We determined the impact of a carbohydrate-enriched drink (Nutricia™ preOp®) on body composition and body water in fasting healthy individuals. We have proven that pre-op did not impact either body composition or body water.

## Data Availability

The data used to support the findings of this study are included within the article or are available from the corresponding author upon request.

## Abbreviations

ASA: American Society of Anesthesiologists
ATM: Adipose tissue mass
BCM: Body cell mass
BMI: Body mass index
DBP: Diastolic blood pressure
E/I: Extracellular water to intracellular water ratio
ECW: Extracellular water
ERAS: Enhanced recovery after surgery
ESPEN: European Society for Clinical Nutrition and Metabolism
FTI: Fat tissue index
HR: Heart rate
ICW: Intracellular water
LTI: Lean tissue index
LTM: Lean tissue mas
SBP: Systolic blood pressure
SD: Standard deviation
TBW: Total body water
